# Biomolecular Fingerprint of Crohn's Disease: A Comparative Raman Spectroscopic Study of Blood and Tissue Samples

**DOI:** 10.1002/jbio.70219

**Published:** 2026-01-18

**Authors:** M. Daniyal Ghauri, Maria Victoria Fernandez, Giovanni Santacroce, Sumedha Chanda, Irene Zammarchi, Ivan Capobianco, Sanathana Konugolu Venkata Sekar, Subrata Ghosh, Katarzyna Komolibus, Stefan Andersson‐Engels, Marietta Iacucci, Rekha Gautam

**Affiliations:** ^1^ Biophotonics@Tyndall Tyndall National Institute Cork Ireland; ^2^ APC Microbiome Ireland, College of Medicine and Health University College Cork Cork Ireland; ^3^ School of Physics University College Cork Cork Ireland

## Abstract

Crohn's disease (CD) is typically diagnosed through endoscopy and histological analysis of biopsies. Currently, specific noninvasive biomarkers for accurate diagnosis and monitoring of CD remain unavailable. This study investigates Raman spectroscopy (RS) as a noninvasive diagnostic and monitoring tool for CD. Plasma and biopsy samples from 66 participants (55—CD, 11—controls) were analyzed to identify disease‐specific molecular alterations. RS revealed significant spectral differences (*p* < 0.05) in amino acids, proteins, carbohydrates, and carotenoids. Decreased valine and arginine levels were consistent with mucosal damage and inflammation, supported by reduced carbohydrate signals indicating barrier impairment. Carotenoid depletion reflected oxidative stress‐related inflammation. Correlations between plasma and tissue spectra suggested that plasma RS can reflect local tissue inflammation. Despite the small and heterogeneous cohort, RS detected biochemical shifts toward healthy profiles following biologic therapy. These findings support RS as a minimally invasive approach for detecting inflammation‐associated molecular alterations, holding potential to guide personalized disease management.

## Graphical abstract

Raman spectroscopy is evaluated as a minimally invasive tool for diagnosing and monitoring Crohn's disease. Correlation between plasma and tissue spectra indicates that plasma measurements reflect local intestinal inflammation, while therapy‐associated biochemical shifts toward healthy profiles highlight its potential for disease monitoring and personalized assessment.

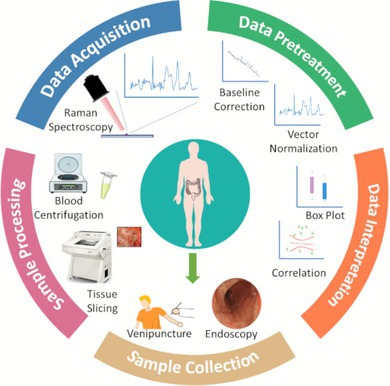

## 1 Introduction

Crohn's disease (CD) is an inflammatory disorder characterized by chronic transmural inflammation along the gastrointestinal tract, most commonly affecting the terminal ileum and colon. Varying incidence and prevalence rates of CD have been reported globally, highlighting its growing public health burden across all ages and ethnic groups [1, 2]. CD typically follows a progressive and destructive course, often leading to complications such as strictures, fistulas, and abscess formation [3]. Patients may remain asymptomatic during the early stages, which necessitates repeated evaluations for adequate decision‐making [4]. Overlapping features with other gastrointestinal disorders further complicate timely and definitive diagnosis, often delaying appropriate management [5]. Such delays may contribute to the development of extra‐intestinal manifestations affecting multiple organ systems, including musculoskeletal, ocular, dermatological, neurological, renal, and metabolic complications [6, 7]. Present diagnostic protocols rely on endoscopy combined with histological examination of tissue biopsies [8]. While these are the gold standard for objective disease assessment, the invasiveness of these procedures, along with the need for bowel preparation and associated risks like perforations, prompts the investigation of novel noninvasive tools [9, 10]. Reliable minimally invasive alternatives could offer safer and more patient‐friendly options for disease monitoring. They can also facilitate implementation of personalized treatment approaches, including Treat‐to‐Target (T2T) strategies, which emphasize achieving and maintaining tight disease control through inflammation reduction, organ preservation, and improved quality of life for CD patients [11].

Blood‐based biomarkers such as C‐reactive protein, albumin, and hemoglobin are promising in this context, as they have shown strong correlations with disease activity indicators and are systemically obtainable with ease [12, 13]. Blood analysis relies on established analytical platforms encompassing techniques such as flow cytometry, mass spectrometry, immunoassays, high‐performance liquid chromatography, and PCR [14, 15, 16, 17, 18]. These methods provide insights into the overall condition of an individual, from nutritional and inflammatory status to immune responses. However, focusing on specific biomarkers can be time‐consuming and may overlook other relevant indicators, especially in a multifactorial condition like CD. Emerging platforms, such as omics technologies, enable comprehensive biological profiling, providing deeper insights into molecular mechanisms driving disease progression [19]. Despite their potential, their widespread clinical adoption remains limited due to the need for micro‐sampling, advanced instrumentation, and complex data analysis pipelines. These factors pose a substantial challenge, especially in low‐resource settings. Thus, developing a rapid, minimally invasive, and accurate diagnostic method is crucial. Identifying reliable biomarkers could streamline diagnosis, reduce dependence on invasive procedures, and improve disease monitoring and treatment decision‐making.

Raman spectroscopy (RS) has emerged as a sensitive and versatile analytical technique capable of label‐free detection of molecular alterations via inelastic light scattering. It enables the simultaneous analysis of multiple biomolecular targets, providing insights into chemical compositional and structural changes within the sample [20]. These capabilities position RS as a promising modality for patient stratification and disease characterization [21]. Its application in inflammatory bowel disease (IBD) has gained momentum, with recent studies demonstrating its expanding role in both metabolic profiling and biomolecular analysis [22, 23, 24, 25]. However, to date, limited research has systematically compared RS profiles from paired tissue biopsies and plasma samples to distinguish between inflamed and non‐inflamed (NI) states in CD. This comparison is especially relevant in CD, where treatment is not curative and often necessitates repeated interventions for tight monitoring. Identification of spectral markers in blood plasma could enable minimally invasive, longitudinal monitoring and guide therapy selection.

This study investigates RS‐based plasma spectral profiles for stratifying CD patients, aiming to evaluate disease activity relative to healthy controls (HCs) and in relation to inflammatory status determined from histological analysis of intestinal biopsies and treatment regimens. To interpret the results, band assignment is made with reference to pure biomolecules. A comprehensive discussion on biological relevance of key biomarkers is presented based on the existing literature.

## 2 Materials and Methods

### 2.1 Chemicals

The following biochemicals were used to facilitate accurate peak assignment: linoleic acid (L1376, Sigma‐Aldrich, USA), l‐α‐phosphatidylserine (840032C, Avanti Polar Lipids LLC., USA), l‐carnitine (T0846, Targetmol Chemicals Inc., USA), indole (I3408, Sigma‐Aldrich), lysine (L5501, Sigma‐Aldrich), l‐tryptophan (T8941, Sigma‐Aldrich), arginine (A5131, Sigma‐Aldrich), valine (V0500, Sigma‐Aldrich), d‐(+)‐xylose (X1500, Sigma‐Aldrich), l‐(−)‐fucose (F2252, Sigma‐Aldrich), collagen (C7661, Sigma‐Aldrich), albumin (A1653, Sigma‐Aldrich), and β‐carotene (C4582, Sigma‐Aldrich).

### 2.2 Sample Collection and Processing

This study involved 66 participants (median age 43.5 years old; male/female 35/31), including 55 CD and 11 HCs, enrolled at Mercy University Hospital and Cork University Hospital in Ireland. CD patients underwent colonoscopy for disease assessment or surveillance, while HCs underwent colonoscopy for gastrointestinal symptoms or colorectal cancer surveillance. Biopsy samples were obtained from the most relevant colonic regions, specifically from areas showing the most significant endoscopic inflammation or those deemed most representative of the underlying diagnosis. Table 1 summarizes the demographic characteristics of the participants. Enrollment was conducted in accordance with the protocol approved by the Clinical Research Ethics Committee of University College Cork. Informed written consent was obtained before collection of blood and tissue samples. Blood was drawn into EDTA‐coated Vacutainer tubes (BD Biosciences) and transported to laboratory premises on ice to minimize degradation. Blood was centrifuged at 10 000 rpm for 10 min (Eppendorf 5415D, Germany), and the plasma was stored at −80°C. Biopsy tissues were also stored at −80°C for further analysis.

**TABLE 1:** Cohort demographics.

Characteristics	CD	Healthy
Enrolment (%)	55 (83.3)	11 (16.7)
Median age^a^ (±SD)	43 (±13.3)	44 (±12.1)
Sex		
Male (%)	31 (56.4)	4 (36.4)
Female (%)	24 (43.6)	7 (63.6)
Therapy		
Advanced therapy (biologics) (%)	34 (61.8)	—
Non‐biologics (%)	14 (25.5)	
Other^b^ (%)	7 (12.7)	—
Histological outcome		
Inflamed (NHI > 1) (%)	24 (43.6)	—
Non‐inflamed (NHI ≤ 1) (%)	26 (47.3)	—
Outcome unknown (%)	5 (9.1)	—

Abbreviation: SD, standard deviation.

^a^ Age at sample collection.

^b^ Combination of surgical intervention, biological, or non‐biological therapy.

### 2.3 Raman Spectroscopy

#### 2.3.1 Blood Plasma

**FIGURE 1:**
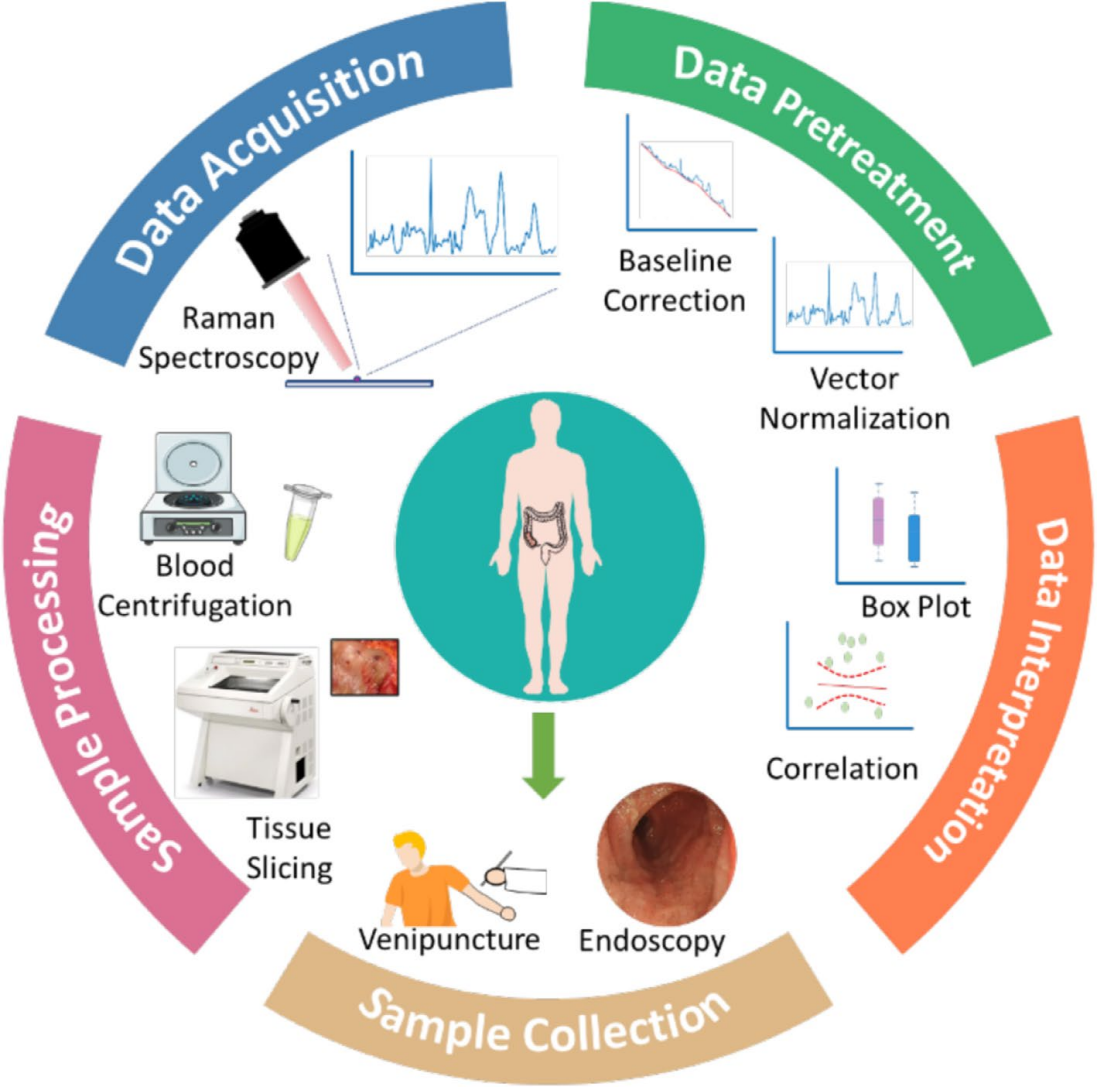
Overview schematic demonstrating the process of sample collection, Raman measurements, and processing.

Microscope slides were prepared by wrapping glass slides (12383118, Fisher Scientific, USA) with aluminum foil and lightly cleaning the top surface with 70% ethanol (CL02.0539, Chem‐Lab, Belgium) soaked wipes (Kimtech Science Kimwipes). Plasma samples were slowly defrosted by transitioning from −80°C to −20°C, then to 4°C, and finally to room temperature. The plasma tube was gently inverted several times to ensure homogeneity. Five 2 μL drops of blood plasma were pipetted onto the slide, spaced approximately 1 cm apart. The slide was then dried inside a plastic desiccator (17250654, Fisher Scientific) connected to a vacuum pump (12911151, Fisher Scientific). Raman measurements were performed using a custom‐built, multi‐configuration Raman system [26]. The system was equipped with a 785 nm laser (Cobolt 08‐01 Series, Sweden), a spectrometer (LS‐785, Teledyne Princeton Instruments, USA), and a back‐illuminated CCD camera (Blaze 400HR, Teledyne Princeton Instruments). Spectra were recorded over 30 s using a 50× objective lens (Nikon CFI60 TU Plan Epi ELWD, Japan), with a 20 mW laser power at the sample. Three random transverse locations within each drop were analyzed, generating a total of 15 spectra per participant. Calibration was performed using neon‐argon lamps, broadband light from the IntelliCal calibration system (Teledyne), and standard spectra of acetaminophen and naphthalene. The sample collection, processing, and data acquisition workflow is illustrated in Figure 1. For Raman bands assignment and interpretation, pure biomolecules were measured in their original form using the same setup and 20 mW power at 785 nm.

#### 2.3.2 Tissue

Biopsy tissues were transferred from −80°C to −20°C cryostat (LEICA CM1950, cryostat, Nussloch, Germany), then cryo‐sectioned into 30 μm slices mounted onto CaF_2_ slides. Sections were fixed in 70% ethanol for 20 min and air‐dried. For each tissue, spectra from 50 spatial points were acquired using a Raman microspectrometer (InVia, Renishaw Inc.) and a motorized *xy*‐sample stage with a 30‐s integration time per location. A 785 nm laser (~20 mW) excited the samples through a 50× long‐working‐distance objective lens. System wavelength calibration was performed before measurements using the silicon Raman line at 520.5 cm^−1^.

### 2.4 Inflammation Assessment

Multiple mucosal biopsies were obtained throughout the colon during endoscopic evaluation. Inflammatory activity was assessed histologically using the Nancy histological index (NHI), where a score of NHI > 1 indicates active (inflamed 'I') disease, and a score of NHI ≤ 1 corresponds to non‐inflamed 'NI' (remission) disease [27]. All specimens were reviewed and scored independently by experienced gastrointestinal pathologists blinded to the clinical data.

### 2.5 Data Preprocessing and Analysis

Preprocessing of the collected Raman spectra for both qualitative and quantitative analyses was performed in MATLAB following the previously described methodology [28]. Key steps included removal of cosmic rays (if present), background correction, noise smoothing, and normalization, as illustrated in Figure 1. Background correction was conducted using the asymmetric least squares method with parameters *λ* = 60 and *p* = 0.0001 for blood plasma and *λ* = 20 000 and *p* = 0.0008 for tissue spectra. Noise smoothing employed a Savitzky–Golay filter with a polynomial order of 2 and a window width of 7. Spectra were then vector normalized. Post‐processing, the spectra (15 for plasma and 40–50 for tissue per participant) were averaged to obtain a single representative spectrum for each individual.

Statistical significance of Raman features was assessed using the Kruskal–Wallis test, with a threshold of *p* < 0.05, and box plots were generated for significant features. Then, to evaluate the association between tissue and blood plasma spectral parameters, Pearson correlation was conducted.

## 3 Results

### 3.1 Raman Spectral Comparison

**FIGURE 2:**
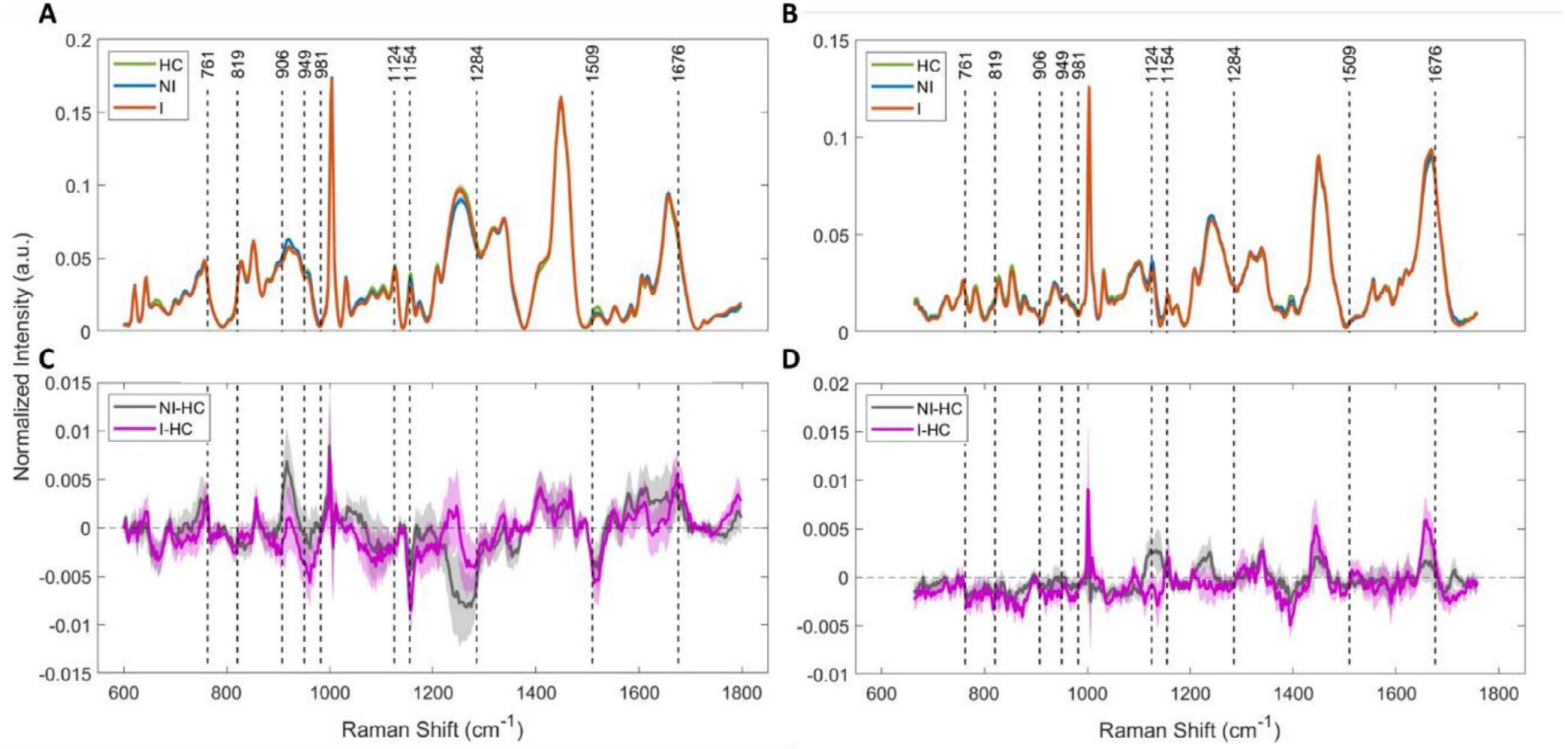
Comparison of averaged Raman spectra of histologically inflamed (I), non‐inflamed (NI), and healthy controls (HC) of (A) plasma and (B) tissue from the same participants. The shaded region represents standard error. (C) and (D) represent the corresponding difference spectra.

This study employed RS to analyze plasma and intestinal biopsy specimens (colon and ileum) from 55 CD patients and 11 HCs. Inflammation stratification was performed based on histological assessments by experienced pathologists. Figure 2A,B present the mean Raman spectra (±standard error “SE”) of plasma and biopsy samples, respectively. Both types of specimens represent a rich source of biomolecules such as proteins, lipids, nucleic acids, metabolites, and signaling molecules [29, 30, 31, 32]. However, given their distinct microenvironments, molecular constituents of both specimens differ substantially, leading to visible spectral variations. This can be seen in the differential analysis comparing CD and HCs in Figure 2C,D. This variation between the two specimen types is likely influenced by the predominance of albumin in plasma and the higher collagen and nucleic acid contributions in tissue, reflecting extracellular matrix components and cellularity [33, 34, 35, 36].

**FIGURE 3:**
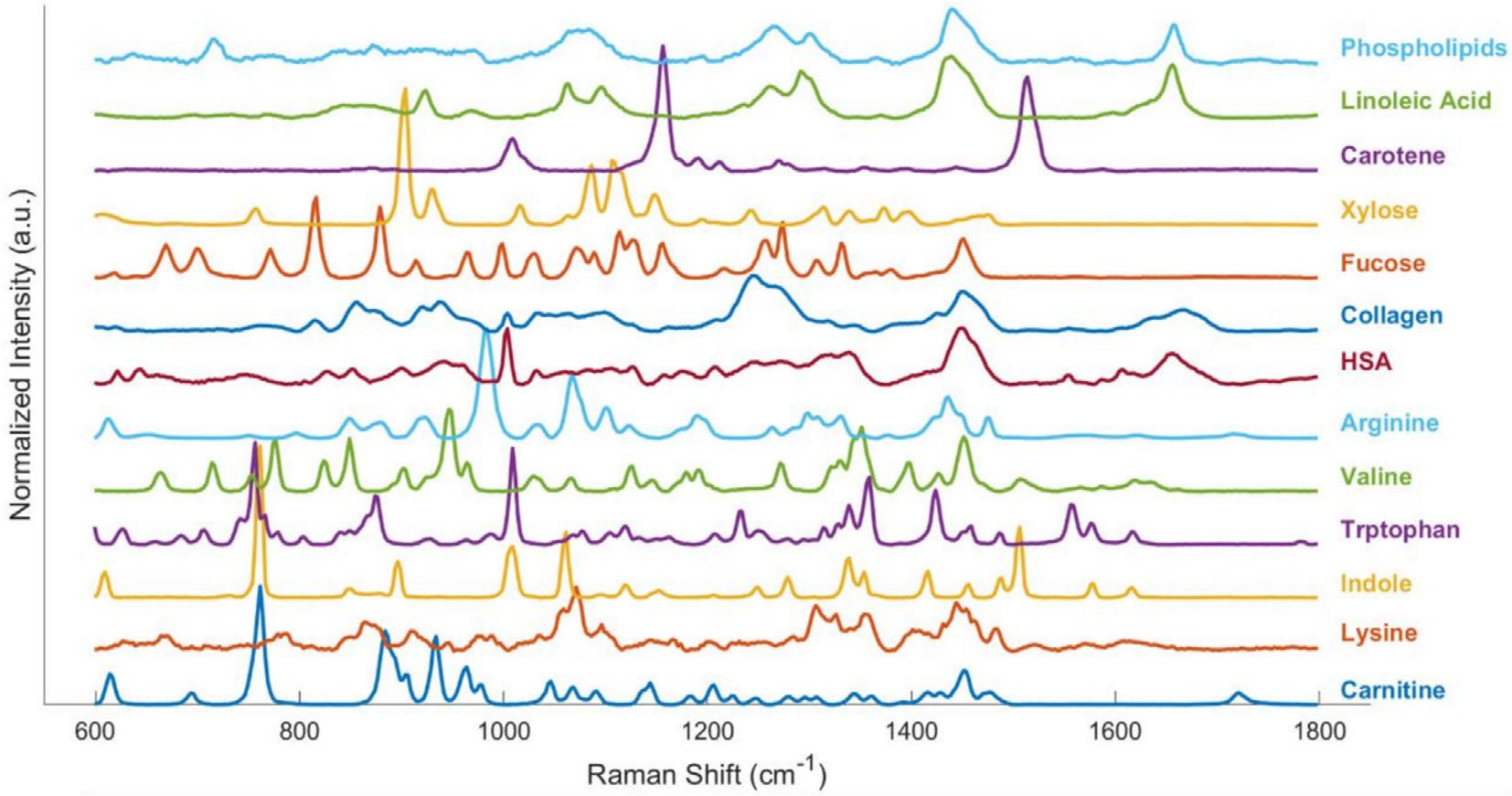
Raman spectra from biomolecules used for the biochemical modeling of plasma and tissue samples of Crohn's disease.

The relevant Raman bands and their assignments were identified based on previously published literature, as summarized in Table 2. To further support qualitative peak assignments and determine which biomolecules are likely to dominate in regions of overlapping bands, Raman spectra were also acquired from isolated chemicals. For these experiments, commercially obtained samples, including various amino acids, carbohydrates, lipids, proteins, and related metabolites, were directly measured using our customized Raman microscope (Figure 3), given prior evidence of their metabolic involvement in IBD [41, 42, 43].

**TABLE 2:** Assignment of most relevant Raman bands [22, 23, 26, 37, 38, 39, 40].

Raman shift (cm^−1^)	Chemical bond assignment	Tentative metabolite association
761	Ring breathing mode, C–N^+^(CH_3_)_3_	Indole, tryptophan, carnitine
819	C–C stretch	Fucose (carbohydrates)
879	C–C stretch, C–O–C ring	Amino acids, carbohydrates
906	C–C and C–O stretch	Xylose (carbohydrates)
949	C–C stretch	Valine (amino acids)
970	═C–H out of plane deformation	Lipids
981	C–C and C–N vibration	Arginine
1067	C–C stretch	Lipids
1124	C–O and C–C stretch, COC glycosidic linkage	Carbohydrates
1154	C–C stretch	Carotenoids
1284	Amide III (C–N stretching and N–H bending)	Proteins
1304	CH_2_ twisting/bending	Lipids
1509	C═C stretch	Carotenoids
1676	Amide I (C═O stretch)	Proteins

### 3.2 Spectral Markers Indicating Molecular Changes

**FIGURE 4:**
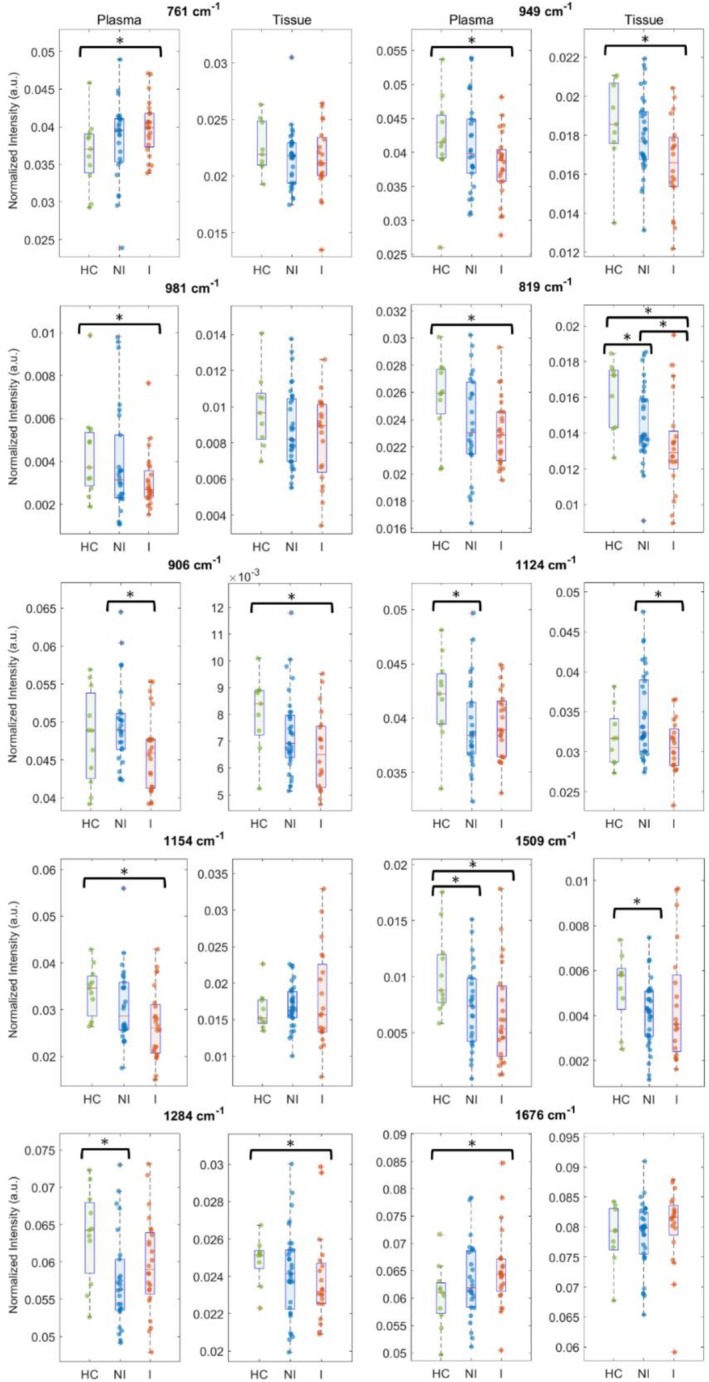
Box plots showing the different Raman intensities of selected peaks with paired samples from both plasma and tissue in healthy control (HC), histologically non‐inflamed (NI) and inflamed (I) participants. Significant differences (*p* < 0.05) are marked by an asterisk.

To further explore the biochemical differences, a comprehensive spectral comparison was conducted across three groups: HC versus CD inflamed (I), HC versus CD NI, and CD I versus NI. Out of 55 CD participants, 24 (43.6%) experienced histologically active inflammation 'I', and 26 (47.3%) were categorized as NI or in remission. To capture all sources of variation during spectral acquisition, a representative spectrum for each participant was obtained by averaging data from five plasma drops and all measurement points across the tissue samples, respectively. These averaged spectra were then subjected to statistical analysis (*p* < 0.05). Figure 4 illustrates several spectral markers identified that effectively differentiate active inflammation from HC and NI cohorts. These markers have been marked in Figure 2 with dotted lines. Overall, RS revealed distinct biochemical alterations in plasma and intestinal tissue from individuals with CD across multiple molecular classes, including amino acids, proteins, carbohydrates, carotenoids, and lipids, as discussed below.

Significant differences were observed in Raman peaks related to amino acids at 761, 949, and 981 cm^−1^. The 761 cm^−1^ band, seen in carnitine and indole ring (Figure 3), showed an increasing trend in CD plasma and a decreasing trend in tissue relative to HCs. The 949 cm^−1^ (valine) and 981 cm^−1^ (arginine) bands showed decreasing trends in both plasma and tissue compared to HCs (Figure 4). In addition, protein‐related Raman modes also differed between groups, with decreased levels of the amide III band (1284 cm^−1^) and an increased level of the amide I band (1676 cm^−1^) in both plasma and tissue of CD participants compared to HCs.

In addition to proteins, significant reductions in carbohydrate‐related Raman bands at 819, 906, and 1124 cm^−1^ were observed in both plasma and tissue of CD participants compared to HCs (Figure 4). These bands also differed significantly between I and NI CD participants. Raman bands at 1154 and 1509 cm^−1^, associated with carotenoids, were also significantly reduced in plasma as compared to HCs (Figure 4). A similar decreasing trend is also observed in intestinal tissue biopsies at 1509 cm^−1^ (Figure 4). Lipid‐related Raman peaks at 969, 1067, and 1304 cm^−1^ showed decreasing trends in both plasma and tissue in CD participants (Figure S1). Although differences between CD patients and HCs were not statistically significant in blood plasma, the decreasing trend observed in plasma matched the reductions seen in paired tissue samples.

### 3.3 Correlation Between Plasma and Tissue Spectral Markers

**FIGURE 5:**
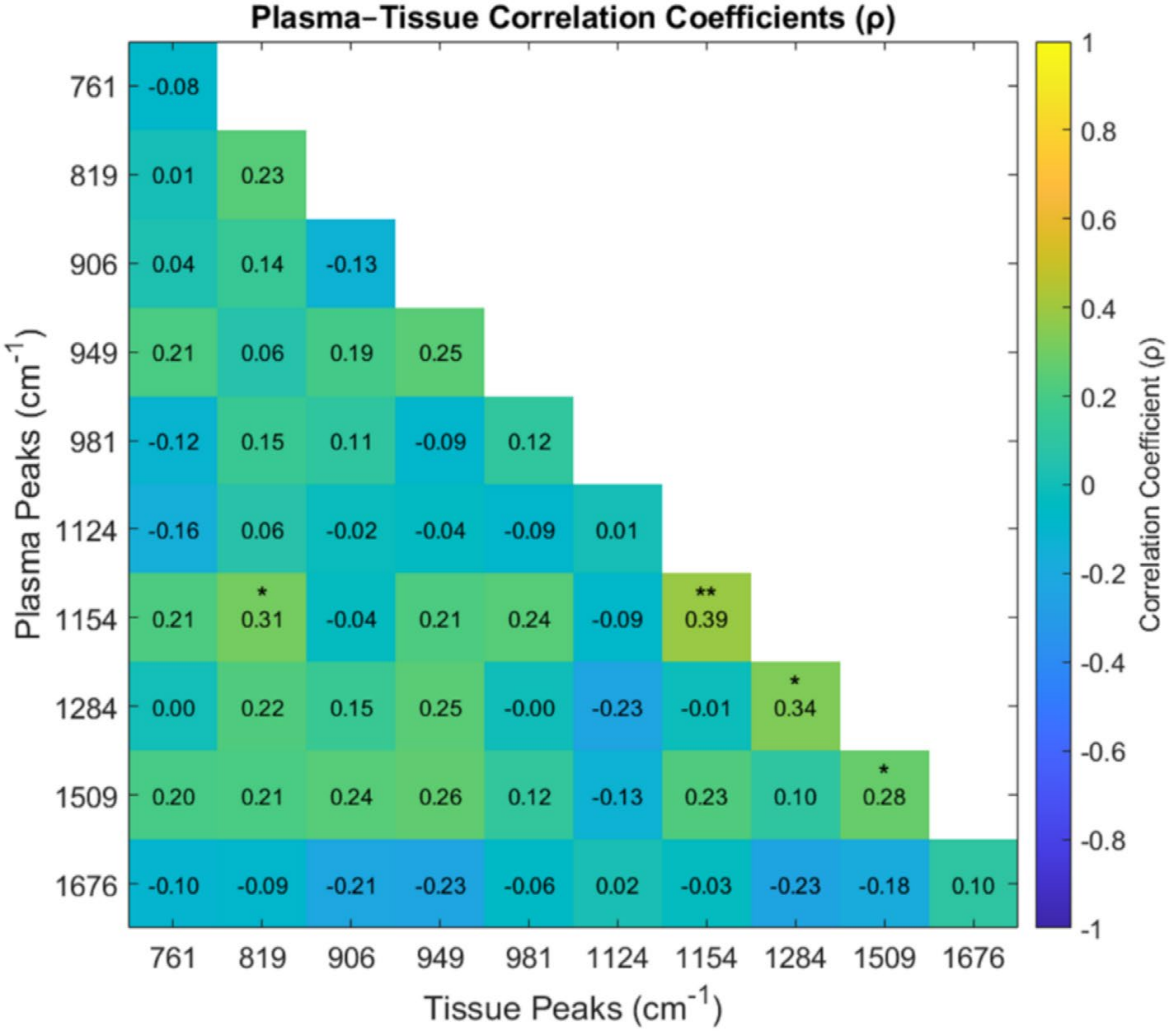
Pearson correlation heatmap showing pairwise associations between plasma and tissue spectral peaks. Only the lower‐left triangle is displayed, with significance levels indicated as *p < 0.05, **p < 0.01, and ***p < 0.005, respectively.

To assess whether systemic spectral changes in plasma reflected local intestinal pathology, correlations between plasma and paired tissue Raman intensities were assessed (Figure 5). The data distribution for each of these peaks is shown in Figure S2. Significant or near‐significant correlations (*p* < 0.05) were observed for peaks at 819, 949, 1154, 1509, and 1284 cm^−1^ representing carbohydrates, valine, carotenoids, and protein structure. No significant correlations were observed for peaks at 761, 981, 906, 1124, and 1676 cm^−1^.

### 3.4 Therapy Monitoring

**FIGURE 6:**
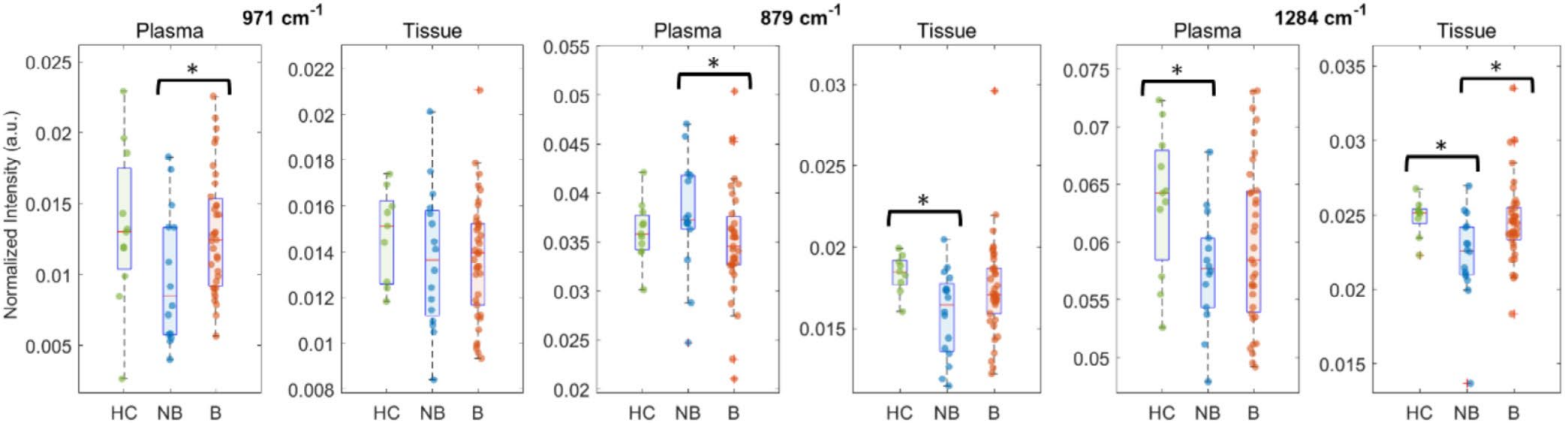
Box plots showing the different Raman intensities of selected peaks with paired samples from both plasma and tissue in HC, non‐biological therapy (NB), and advanced therapy (biologics “B”) administered participants. Significant differences (*p* < 0.05) are marked by an asterisk.

Spectral profiles of patients receiving advanced biologic therapy were compared with those not receiving biologics. Significant differences were identified at 971 cm^−1^ (lipids), 879 cm^−1^ (amino acids and carbohydrates), and 1284 cm^−1^ (proteins). In biologically treated patients, Raman intensities at these peaks approached those of HCs (Figure 6). Other spectral features showed no statistically significant treatment‐related differences (Figures S3 and S4).

## 4 Discussion

This study demonstrates that RS can detect distinct biochemical alterations in both plasma and intestinal tissue of individuals with CD, revealing consistent changes across amino acids, proteins, carbohydrates, carotenoids, and lipids. These findings highlight the multisystem metabolic disturbances characteristic of CD and underscore the potential of RS as a minimally invasive tool for monitoring disease activity and therapeutic response.

Alterations in amino acid‐associated Raman features were among the notable findings (Figure 4). Increases in the 761 cm^−1^ band in plasma, with a decreasing trend in tissue, may reflect disruptions in carnitine handling or inflammation‐driven shifts in tryptophan/indole metabolism. Altered tryptophan–indole metabolism is also consistent with prior observations in CD [37]. The reductions observed in valine (949 cm^−1^) and arginine (981 cm^−1^) align with reported depletion of branched‐chain amino acids and arginine in active CD [44, 45, 46, 47]. These changes likely reflect increased metabolic demand, mucosal damage, inflammation‐driven catabolism, and impaired absorption. Notably, valine is lower in CD than in ulcerative colitis, highlighting its potential as a disease‐specific biomarker [46]. The observed protein structural changes (decreased amide III and increased amide I levels) are consistent with inflammation‐driven protein unfolding, proteolysis, collagen remodeling, and elevated matrix metalloproteinase activity in penetrating CD phenotypes [48]. Changes in plasma proteins may further reflect hypoalbuminemia commonly reported in CD [49, 50].

Significant reductions in carbohydrate‐related Raman bands (819, 906, and 1124 cm^−1^) likely reflect degradation of the intestinal mucin layer and impaired glycosylation (Figure 4). Reduced fucose‐related signals are consistent with FUT2 polymorphisms that impair intestinal fucosylation and compromise mucosal barrier function [51, 52]. Similarly, reduced xylose‐associated signals reflect malabsorption linked to intestinal inflammation and bacterial overgrowth [53, 54]. Together, these findings highlight the critical role of carbohydrate metabolism in gut barrier integrity and immune regulation. Along with carbohydrates, lipids also play key roles in immune regulation and gut health, often through interactions with the gut microbiota. Although lipid‐related Raman peaks showed nonsignificant changes in plasma, the consistent reductions observed in intestinal tissue suggest altered local lipid metabolism (Figure S1). Dysregulation of microbiota‐derived fatty acids, together with inflammation‐associated lipid malabsorption, may contribute to these tissue‐level changes [55, 56, 57].

Carotenoid‐associated bands (1154 and 1509 cm^−1^) were also depleted (Figure 4), consistent with the presence of elevated oxidative stress in CD [37, 58]. Enhanced reactive oxygen species production from activated immune cells, combined with reduced dietary antioxidant availability due to malabsorption, likely contributes to this depletion [59, 60]. These results demonstrate the potential of RS as a tool for noninvasive monitoring of oxidative burden in IBD.

A key strength of this study is the paired analysis of plasma and tissue samples. The observed correlations between plasma and tissue Raman markers indicate that several circulating biochemical features reflect local inflammatory alterations within the intestinal mucosa (Figure 5 and Figure S2). This suggests that plasma RS has potential as a minimally invasive proxy for mucosal inflammatory activity. However, the absence of correlations for certain peaks also highlights intrinsic compositional differences between plasma and tissue.

Biologic therapies, including anti‐TNF agents such as infliximab and anti‐integrins like vedolizumab, target key inflammatory pathways to induce and maintain remission in moderate‐to‐severe CD [61]. These treatments reduce mucosal injury, prevent complications such as fistulas, and improve quality of life. The recovery of lipid, protein, and amino acid/carbohydrate Raman markers toward healthy profiles in biologic‐treated patients supports the potential of RS for monitoring therapeutic response (Figure 6 and Figure S3). These findings are consistent with reports of metabolic normalization accompanying mucosal healing [62]. However, given the limited sample size and mixed biologic regimens in this cohort, these results should be viewed as preliminary. Larger studies are needed to determine the specific effects of individual biologic agents.

### 4.1 Limitations

Our results may contribute to the development of minimally invasive approaches for both diagnosis and monitoring of CD. While the findings are encouraging, several limitations must be acknowledged. Most notably, the relatively small sample size, particularly within the HC group, represents a constraint which may affect the statistical robustness of the findings. On a similar note, systematic evaluation of confounding factors such as diet, medication, physical activity, and lifestyle on blood metabolic profiles will further strengthen the hypothesis of using RS's unique molecular sensitivity for CD.

Patients on biologics in this study were undergoing treatment with various therapies, including adalimumab, vedolizumab, and ustekinumab. However, due to the limited sample size, these patients were analyzed as a single group, precluding a detailed assessment of drug‐specific metabolic signature. Future studies with larger longitudinal cohorts will be essential to look into the effects of individual biologics on blood metabolite profiles. Such analyses could contribute to the development of personalized therapeutic strategies by identifying metabolic signatures predictive of response to specific biologic agents.

## 5 Conclusion

This study demonstrates the potential of RS to detect plasma spectral markers reflecting molecular changes associated with intestinal inflammation in CD patients. This was further validated through correlation with histological analysis of intestinal tissue biopsies. Raman band assignments and interpretations were made based on measurements of pure biomolecules. The spectral markers identified in this study align with molecular profiles previously reported in multi‐omics studies of CD, strengthening the biological relevance of our findings. The rapid molecular‐level layer of diagnostic insights offered by RS presents a promising supporting modality to the current gold standard approaches for CD. Its minimally invasive nature is particularly advantageous for patients who require frequent assessments, offering a more comfortable approach for longitudinal follow‐up. Future work should aim to expand the patient cohort and validate these findings in a larger cohort.

## Funding

This work was supported by H2020 Marie Skłodowska‐Curie Actions (860185), SEFS Tyndall New Connections Grant and Science Foundation Ireland (SFI/15/RP/2828, SFI‐12/RC/2276 P2, and SFI‐22/RP‐2TF/10293).

## Conflicts of Interest

The authors declare no conflicts of interest.

## Supporting information


**Figure S1:** (A) Box plots showing the different Raman intensity of lipid‐specific Raman peaks with paired samples from both plasma and tissue in HC, histologically inflamed (I), and non‐inflamed (NI) participants. Significant differences (*p* < 0.05) are marked by an asterisk (B) Correlations between plasma and paired tissue for each participant. The dotted lines indicate the 95% confidence intervals. Correlation coefficients and *p* values for each intensity ratio are displayed in the individual panels.
**Figure S2:** Correlations between plasma and tissue pairs for each participant. Dotted lines indicate the 95% confidence intervals. Correlation coefficients and *p* values for each intensity ratio are displayed in the individual panels.
**Figure S3:** Comparison of averaged Raman spectra of patients on advanced therapy (Biologics ‘B’), non‐biological therapy (NB), and healthy controls (HC) of (A) plasma and (B) tissue from the same participants. The shaded region represents standard error. (C) and (D) represent the corresponding difference spectra.
**Figure S4:** Box plots showing the different Raman intensity of selected peaks with paired samples from both plasma and tissue in HC, non‐biological therapy (NB), and advanced therapy (biologics ‘B’) administered participants. Significant differences (*p* < 0.05) are marked by an asterisk.

## Data Availability

The data that support the findings of this study are available on request from the corresponding author. The data are not publicly available due to privacy or ethical restrictions.
