# Supplementary material for: Biomolecular Fingerprint of Crohn's Disease: A Comparative Raman Spectroscopic Study of Blood and Tissue Samples
Source: J Biophotonics. 2026 Jan 18;19(1):e70219. doi: 10.1002/jbio.70219 (PMC12812440; doi:10.1002/jbio.70219)
Supplement: Supplementary file 1 — Figure S1: (A) Box plots showing the different Raman intensity of lipid‐specific Raman peaks with paired samples from both plasma and tissue in HC, histologically inflamed (I), and non‐inflamed (NI) participants. Significant differences (p < 0.05) are marked by an asterisk (B) Correlations between plasma and paired tissue for each participant. The dotted lines indicate the 95% confidence intervals. Correlation coefficients and p values for each intensity ratio are displayed in the individual panels. Figure S2: Correlations between plasma and tissue pairs for each participant. Dotted lines indicate the 95% confidence intervals. Correlation coefficients and p values for each intensity ratio are displayed in the individual panels. Figure S3: Comparison of averaged Raman spectra of patients on advanced therapy (Biologics ‘B’), non‐biological therapy (NB), and healthy controls (HC) of (A) plasma and (B) tissue from the same participants. The shaded region represents standard error. (C) and (D) represent the corresponding difference spectra. Figure S4: Box plots showing the different Raman intensity of selected peaks with paired samples from both plasma and tissue in HC, non‐biological therapy (NB), and advanced therapy (biologics ‘B’) administered participants. Significant differences (p < 0.05) are marked by an asterisk. [file JBIO-19-e70219-s001.docx]

# SUPPLEMENTARY INFORMATION

## Fig. S1


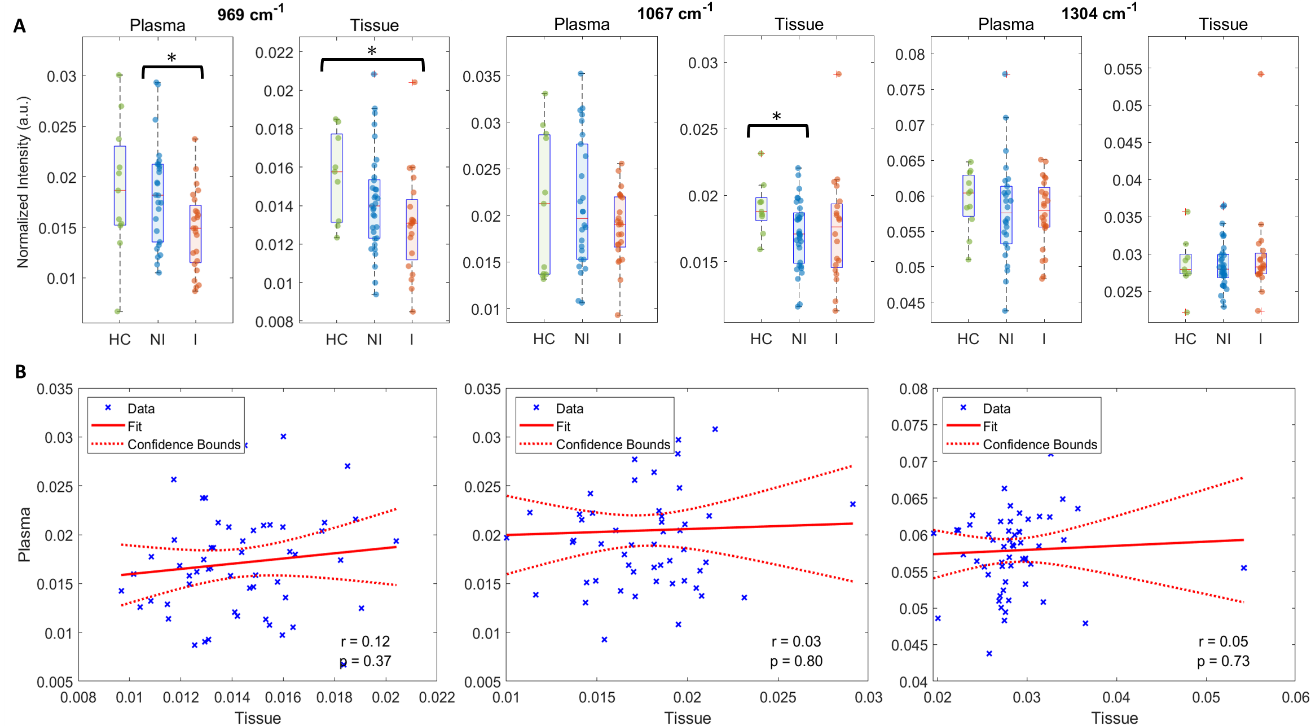


Fig. S1: (A) Box plots showing the different Raman intensity of lipid-specific Raman peaks with paired samples from both Plasma and Tissue in HC, histologically inflamed (I) and non-inflamed (NI) participants. Significant differences (p<0.05) are marked by an asterisk (B) Correlations between plasma and paired tissue for each participant. The dotted lines indicate the 95% confidence intervals. Correlation coefficients and P values for each intensity ratio are displayed in the individual panels.

## Fig. S2


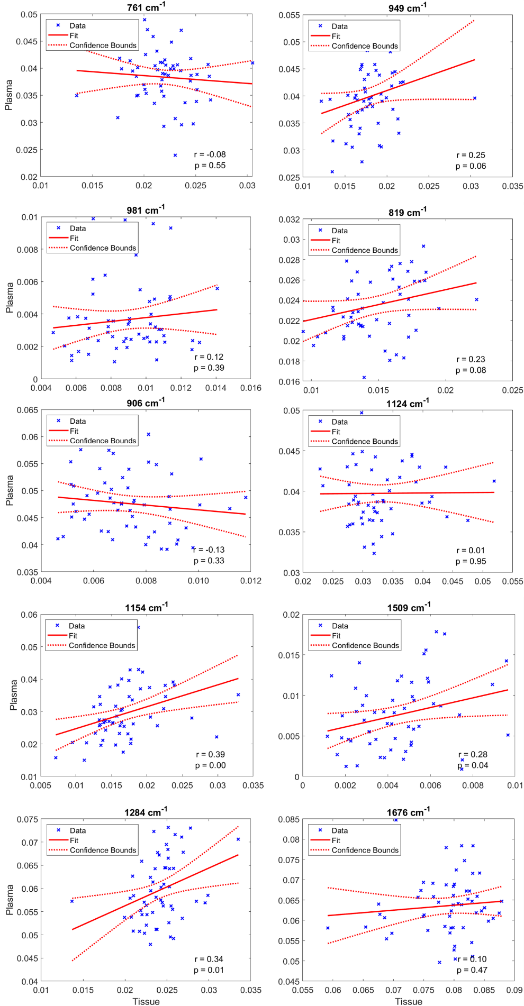


Fig. S2: Correlations between plasma and tissue pairs for each participant. Dotted lines indicate the 95% confidence intervals. Correlation coefficients and p values for each intensity ratio are displayed in the individual panels.

## Fig. S3


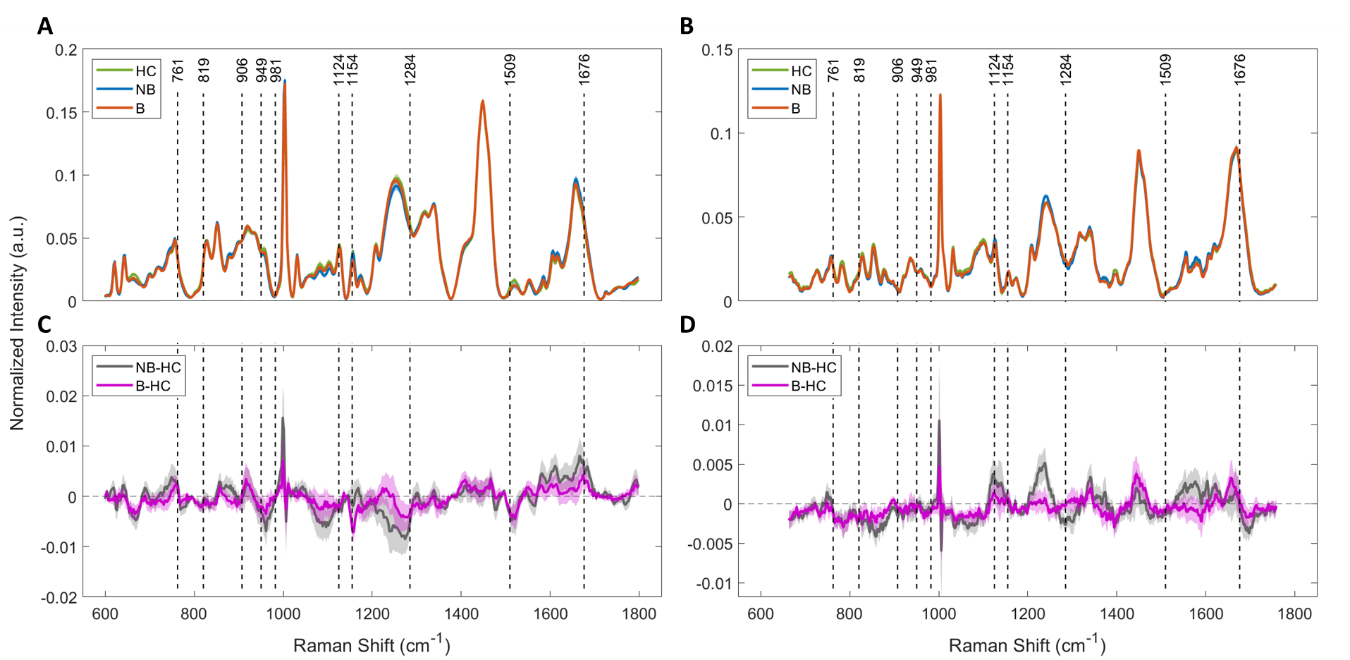


Fig. S3: Comparison of averaged Raman spectra of patients on advanced therapy (Biologics ‘B’), non-biological therapy (NB) and healthy controls (HC) of (A) Plasma and (B) Tissue from the same participants. The shaded region represents standard error. (C) and (D) represent the corresponding difference spectra.

## Fig. S4


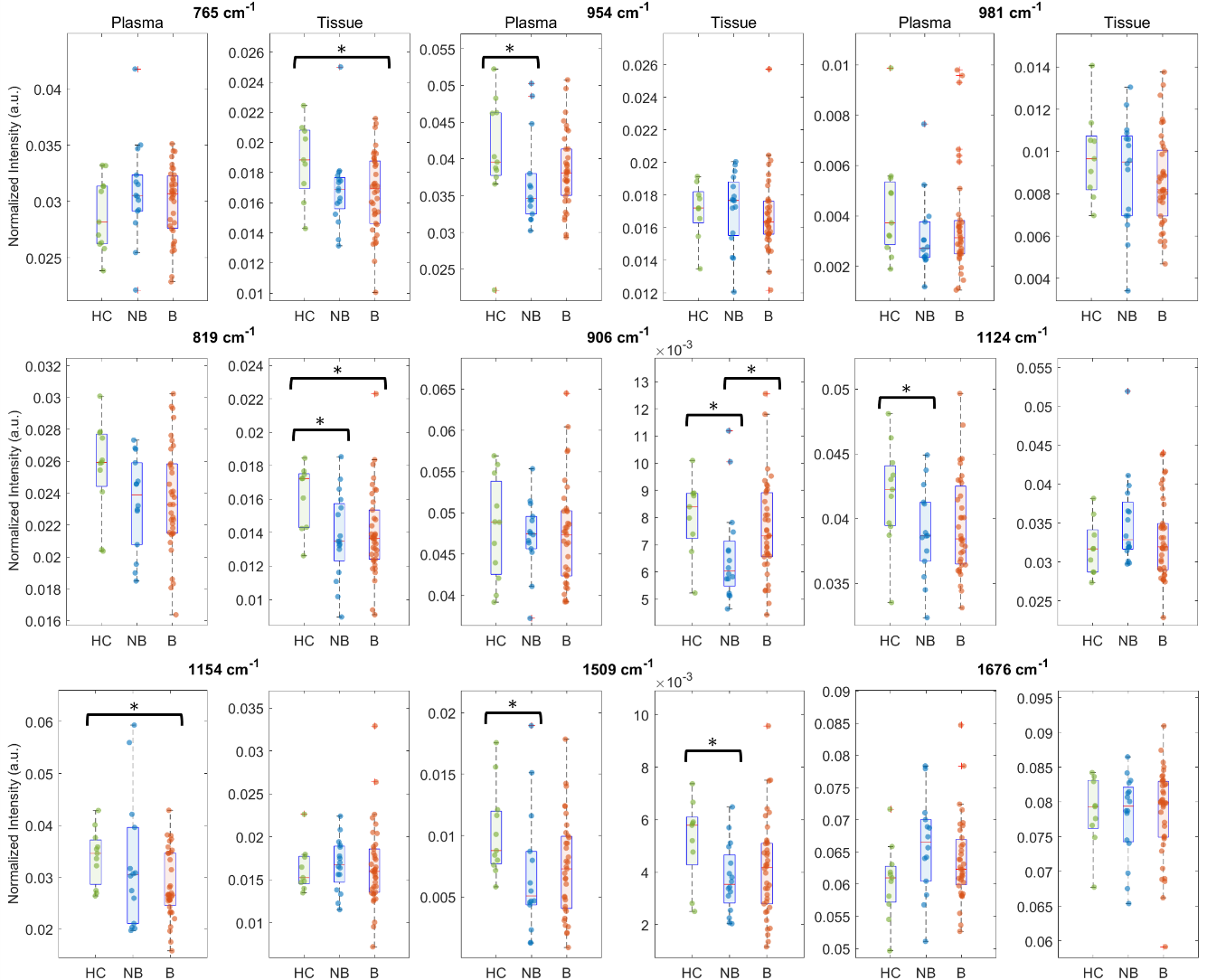


Fig. S4: Box plots showing the different Raman intensity of selected peaks with paired samples from both Plasma and Tissue in HC, non-biological therapy (NB) and advanced therapy (Biologics ‘B’) administered participants. Significant differences (p<0.05) are marked by an asterisk.
